# A Short–Time Scale Colloidal System Reveals Early Bacterial Adhesion Dynamics

**DOI:** 10.1371/journal.pbio.0060167

**Published:** 2008-07-08

**Authors:** Christophe Beloin, Ali Houry, Manuel Froment, Jean-Marc Ghigo, Nelly Henry

**Affiliations:** 1 Unité de Génétique des Biofilms, Institut Pasteur, CNRS URA 2172, Paris, France; 2 Laboratoire Physico-Chimie Curie, Institut Curie, CNRS UMR 168, Université Paris VI , Paris, France; Imperial College London, United Kingdom

## Abstract

The development of bacteria on abiotic surfaces has important public health and sanitary consequences. However, despite several decades of study of bacterial adhesion to inert surfaces, the biophysical mechanisms governing this process remain poorly understood, due, in particular, to the lack of methodologies covering the appropriate time scale. Using micrometric colloidal surface particles and flow cytometry analysis, we developed a rapid multiparametric approach to studying early events in adhesion of the bacterium Escherichia coli. This approach simultaneously describes the kinetics and amplitude of early steps in adhesion, changes in physicochemical surface properties within the first few seconds of adhesion, and the self-association state of attached and free-floating cells. Examination of the role of three well-characterized E. coli surface adhesion factors upon attachment to colloidal surfaces—curli fimbriae, F-conjugative pilus, and Ag43 adhesin—showed clear-cut differences in the very initial phases of surface colonization for cell-bearing surface structures, all known to promote biofilm development. Our multiparametric analysis revealed a correlation in the adhesion phase with cell-to-cell aggregation properties and demonstrated that this phenomenon amplified surface colonization once initial cell-surface attachment was achieved. Monitoring of real-time physico-chemical particle surface properties showed that surface-active molecules of bacterial origin quickly modified surface properties, providing new insight into the intricate relations connecting abiotic surface physicochemical properties and bacterial adhesion. Hence, the biophysical analytical method described here provides a new and relevant approach to quantitatively and kinetically investigating bacterial adhesion and biofilm development.

## Author Summary

When bacteria grow on solid surfaces, they can form three-dimensional communities called biofilms. Within these complex structures, bacteria can develop specific tolerance to different microbiocides, causing serious health and economic problems. Investigations of the key molecular events involved in biofilm formation have shown that surface-exposed adhesin proteins promote this process, but many questions remain regarding the mechanisms and biophysics of surface adhesion. We introduced an original approach to investigating the very early steps in bacterial adhesion that uses dispersed colloidal surfaces as microbial adhesion substrates. Using flow cytometry, we performed a quantitative real-time analysis of adhesion kinetics of several strains of the bacterium Escherichia coli, which were genetically engineered to produce well-characterized cell-surface adhesins that are known to promote biofilm development. We provide evidence for previously unknown adhesin-dependent behaviors, such as clear-cut differences in the very initial phases of surface colonization. We also demonstrate that initial adhesion correlates with almost instant surface property changes, and that cell-to-cell association might serve as an amplification mechanism for surface colonization. We therefore provide a new understanding of the intricate relationships between the physico-chemistry of abiotic surfaces and bacterial adhesion.

## Abstract 3

Combining micrometric colloidal beads as adhesion substrates with flow cytometric analysis allows for a high-resolution analysis that reveals adhesin-dependent behavior in the very first steps of surface colonization by bacteria.

## Introduction

Bacterial growth on surfaces and interfaces leads to the formation of three-dimensional communities called biofilms [1]. Biofilm-specific tolerance to different biocidal treatments used in health care facilities stimulated the investigation of key molecular events in biofilm formation, and molecular factors promoting bacterial adhesion have been characterized, including surface-exposed adhesins and polysaccharidic polymers. However, initial cell surface attachment itself is still poorly understood, and many questions remain regarding biophysical aspects of the adhesion process due to lack of appropriate investigation tools [2,3].

Physico-chemical approaches based on the Derjaguin-Landau-Verwey-Overbeek (DLVO) [4] theory, although reliable in predicting interactions between well-controlled model hard spheres, have often proven inappropriate for modeling bacterial adhesion [5]. This is likely due to the multiplicity of parameters involved in the adhesion process, influenced both by biological and environmental factors. On the other hand, most biological approaches to surface colonization rely on procedures that develop on an hourly or daily scale. Hence, interpretations are often made at the final adsorption stage or at equilibrium, when initial adhesion is blurred by subsequent biofilm development steps, thus preventing precise kinetic analysis of the adhesion process [3]. Alternative short-term approaches are therefore needed to provide quantitative and kinetic information on the early stage of bacterial interaction with a surface.

We introduced a new methodology which takes advantage of advanced flow cytometry (FCM) analyses [6] to characterize initial bacterial cell surface attachment. Only a few studies monitoring bacterial attachment to mammalian cells using FCM have been published [7,8]. Thus far, stream techniques involved in FCM have appeared incompatible with the study of bacterial adhesion usually studied on macroscopic plane surfaces. We have overcome this apparent antagonism by choosing dispersed surfaces in the form of well-characterized micrometric particles as adhesion substrates in order to explore initial events of surface colonization by the bacterium Escherichia coli. We used charged particles as adhesion substrates, representing the most widespread situation for surfaces immerged in aqueous media due either to their inherent ionization state or to the water ion structure at the interfaces. Then, we examined the contribution of three well-characterized E. coli adhesion factors with a time resolution of a few seconds and the precision of one bacterium per particle. This revealed several phases leading to surface colonization, and evidenced clear-cut differences strongly dependent on the nature of the adhesin expressed at the cell surface. We also explored the contribution of cell-to-cell aggregation properties and particle surface physicochemical property changes throughout the colonization process. This analytical procedure therefore opens up new perspectives in the understanding of bacterial adhesion to abiotic surfaces.

## Results

### Description and Characterization of the Micrometric Colloidal System

**Figure 1:**
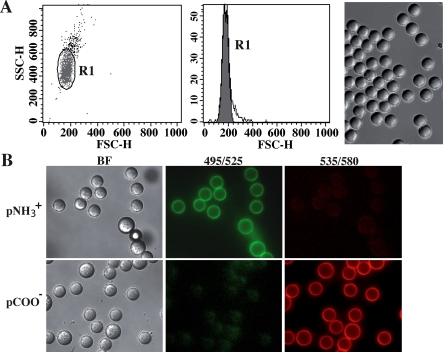
Colloidal Substrates (A) FCM scattering plot and corresponding bright field microscope images of a COO^–^ particle sample washed in water and suspended in PBS. 80% of events were concentrated in the scattering value region R1 corresponding to a monodispersed, 10-μm-diameter population. Outliers corresponded to a population of slightly larger particles and a few doublets. Cationic (NH_3_^+^) particles displayed the same scattering characteristics as anionic (COO^–^) particles. (B) Microscopic images of PYR- (2nd column) and PI- (3rd column) labeled cationic (1st row) and anionic (2nd row) particles. Dye concentration equal to 16.7 nM.

To develop a short–time scale method for studying early bacterial adhesion, we used spherical micrometric colloidal particles as adhesion substrates, both positively charged (aminated, NH_3_^+^) and negatively charged (carboxylated, COO^–^) with zeta potentials of +45 mV and −55 mV, respectively. Suspensions of 10-μm particles were resuspended in phosphate-buffered saline (PBS) and examined by microscopy and FCM. 80% of the events detected with the two types of particles were concentrated in scattering value region R1 (free particles scattering region), corresponding to a monodispersed population (Figure 1A and unpublished data).

Particle surface charge properties were traced using an anionic dye (pyranine, PYR) and a cationic dye (propidium iodide, PI), the fluorescent labeling of which was detected by microscope imaging (Figure 1B) and FCM (Figure S1). Both probes displayed fast (adsorption half-time < 1 s) and selective electrostatic adsorption onto particles carrying opposite charges. PYR was emitted in the FL1 channel [mean fluorescence intensity in channel 1] (525 ± 10 nm) and PI in the FL3 channel [mean fluorescence intensity in channel 3] (>670 nm). Labeling properties were conserved in M63B1 medium, the rather high ionic strength (≈200 mM versus 150 mM,e.g., for PBS) medium used in this study both for growth of the bacteria and the adhesion assay (see below), indicative of strong and irreversible association, which demonstrated that the chosen pairs of dyes were flexible surface-charge reporters in the heterogeneous context of a bacterial suspension.

### FCM Monitoring of Isolated and Aggregated Bacteria in Suspension

**Figure 2:**
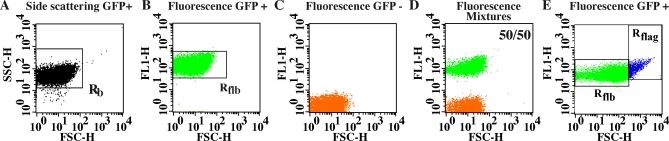
Bacterial FCM Scattering (A) and fluorescence dot plots of all GFP-labeled (B), all non GFP-labeled cells (C) and a mixture (50/50) of both (D). Aliquots of exponentially growing cultures were diluted in PBS to obtain a 5 × 10^6^ cells/ml suspension. (E) FL1 dot plots of a slightly aggregated cell population (R_flag_, right plot); in this case, 3% of the objects are considered aggregates.

To determine optimal recording conditions for bacterial adhesion in the colloidal system, we analyzed samples composed of planktonic cultures of fluorescent and non-fluorescent E. coli K-12 strains MG1655 and MG1655*gfp*, respectively, mixed at various proportions. Due to their smaller size, bacteria had to be analyzed with higher scattering photomultiplier gains than colloidal particles and appeared in a distinct acquisition window. We isolated a bacterial signal in scattering region R_b_ containing at least 98% of the recorded events** (** Figure 2A**)**. Depending on sample composition, fluorescence plots FL1/forward scattering (FSC) stemming from R_b_ events displayed one or two subsets, each with a characteristic mean fluorescence FL1 value conserved in the whole sample series **(** Figure 2B–2D**)**. The ratio of the number of cells counted in high and low fluorescence subsets accurately matched values expected from the mixtures (Figure S2), indicating that the recorded signals originated from single cells. Indeed, if more than one cell was detected in a single event, then higher number of subsets depending on the number of detected cells would have appeared with intermediate fluorescence intensities. This enabled us to determine a cell unitary fluorescence value *fl*_i_ from the mean FL1 fluorescence of an R_flb_ gate (bacteria region) containing the fluorescent bacteria (Figure 2B). When green fluorescent protein (GFP)-labeled cells were allowed to rest at room temperature for 2 h, we observed the formation of cell aggregates that produced higher scattering and a fluorescence signal defining a new gate, R_flag_ (bacterial aggregates fluorescence (channel 1) region in bacteria records) (Figure 2E). The aggregate scattering parameters were sufficiently different from those of colloidal particles to avoid any confusion between the two types of objects even when aggregate sizes increased. Therefore, the R_flag_ number of events (N(R_flag_)), mean fluorescence (FL1(R_flag_)), and mean forward scattering (FSC(R_flag_)) enabled us to report the dispersion state of the cell suspension.

### Quantitative and Qualitative Multiparametric Monitoring of Cell Adhesion Events in a Colloidal Suspension

**Figure 3:**
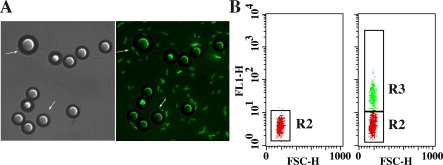
Bacterial Adhesion in Colloidal Suspension (A) Microscope images of MG1655*gfp E. coli* cells and particles brought into contact with particles in M63B1 medium for 20 min. Bright-field and bright-field fluorescence combined images are shown. Adhesive events can be observed on the combined image; examples are indicated by white arrows. (B) Corresponding fluorescence dot plots are shown for particles alone (left) and cells and particles brought into contact for 20 min (right). Gate R2 corresponds to bare particles, whereas gate R3 corresponds to colonized particles.

To study bacterial adhesion onto colloidal substrates, GFP-labeled E. coli cells were brought into contact with cationic particles under gentle stirring, producing mild shear stress on the order of a few tens of piconewtons per contact. While this stirring abolished gravity effects, it also screened cell motility. Therefore, although bacterial motility has been shown to play a role in surface colonization under static conditions [9], its effect is here masked by velocity gradients due to stirring-induced hydrodynamic shear. The question of the role of bacterial self-propulsion through flagellar motility is thus not addressed here. The FCM signal was recorded on aliquots taken at different times from the cell-particle sample using a sample flow speed of 1 μl/s and an acquisition time equal to 5 s. Direct microscopic observation showed that bacteria adhered to colloidal particles, leading to the emergence of a new cluster of higher fluorescence on particle FL1/FSC cytograms, corresponding to particles carrying at least one bacterial cell (Figure 3A and B). Bare (R2) and colonized particles (R3) were thus easily discriminated on the basis of their fluorescence intensity and described by the two particle subsets stemming from the R1 gate (Figure 3B). As shown in an experiment performed using a 200-fold excess of colloidal particles over bacteria (Figure S3)—which ensured that only rare but single cell adhesion events would statistically appear—bacterial fluorescence was not affected by adhesion to particles. Therefore, the mean number of particle-bound bacteria (*n*_F_) could be obtained simply by dividing mean particle fluorescence (FL1(R1)) by bacterial unitary fluorescence (*fl*_i_ = FL1(R_flb_)).

Adhesion in the colloidal system could be regarded as the sum of discrete efficient collisions occurring between one particle and one cell, and could then be adequately described by Poisson's law as follows:

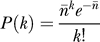
where *P*(*k*) was the probability of finding a particle bearing *k* bacteria when the mean of the distribution was 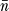 . For a given acquisition, the experimental value of 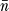 was *n*_F_. *f*_c_, the colonized particle fraction, was experimentally given by *f*_c_ = N(R3)/[N(R2) + N(R3)] and was linked to *P*(0), the probability of finding a particle free of bacteria by *f*_c_ *=* (1 *– P*(0)), which gave 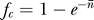 .

For any value of *f*_c_, we could thus calculate the mean of the corresponding statistical distribution *n*_P_ *= –*ln (1 – *f*_c_)*.* This provided a direct means of characterizing adhesion distribution and detecting eventual cooperative effects by comparing *n*_P_ to experimental value *n*_F_ calculated from mean fluorescence. This also enabled the definition of a cooperative index λ, given by the ratio *n*_F_/*n*_P_.

To achieve a complete description of the cell particle suspension, 5 μl of a 10^−6^ M PI solution were added to each aliquot taken from the incubation sample. Labeling performed just before the FCM analysis and after the cell–particle interaction allowed us to characterize the particle surface charge. It did not, however, affect cell–particle association, as checked by parallel recordings of unlabeled and labeled samples (unpublished data).

The complete analysis enabled us to determine, for each sample test, the free-floating cell aggregation state, the cell particle surface association degree, as well as the particle surface charge state. It also reported the potential cooperative character of cell to particle surface binding.

The colloidal system approach therefore enabled full quantification of the early bacterial adhesion process. Moreover, the sensitivity of signal recording enabled us to determine adhesion kinetics taking place within the range of few seconds after cell–particle contact.

### Curli Expression Alters the Kinetics of Initial Attachment of Bacteria to an Abiotic Surface

**Figure 4:**
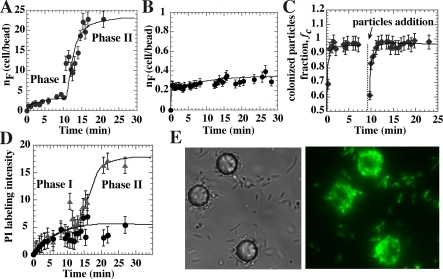
Effect of Curli Production on Early Surface Adhesion Adhesion kinetic curves of MG1655*gfpompR234* (A) and MG1655*gfp*Δ*csgA* (B). Particles and cells were brought into contact at time *t* = 0. Cell to particle ratio was around 200 and particle concentration equal to 6 × 10^6^/ml. (C) Colonized particle fraction kinetics are shown for two sequential additions of fresh NH_3_^+^ particles to MG1655*gfpompR234* cells. 50 μl of stock particles were added to a cell/particle sample after 9 min incubation once the first colonization phase leveled off. (D) Charge inversion of NH_3_^+^ particle during surface colonization. FL3 intensity in time due to PI adsorption (2 × 10^−8^ M) is shown for MG1655*gfpompR234* (▴) and MG1655*gfp*Δ*csgA* (•). (E) Bright field and fluorescence microscope images of MG1655*gfpompR234* engaged in second-phase colonization of NH_3_^+^ particles.

Curli are thin aggregative fimbriae assembled at the cell surface of most Enterobacteriaceae, in which they have been shown to promote adhesion to abiotic surfaces [10]. They form 6–12-nm-diameter structures whose length varies between 0.5 and 1 μm [11]. To explore the contribution of curli to early steps in bacterial adhesion, we used fluorescent E. coli either overexpressing curli due to a mutation in *ompR*, the positive regulator of curli expression (MG1655*gfpompR234*) [10], or deprived of curli (MG1655*gfp*Δ*csgA*). We compared the adhesion profiles of mid-exponential phase growth cultures on aminated particles using a cell-to-particle ratio close to 200. FCM analyses of 10 μl aliquots taken from both cultures at ∼30 s intervals revealed that the presence of curli strongly modified early initial attachment of bacteria to an abiotic surface. The adhesion kinetics of the strain constitutively expressing curli displayed two distinct binding phases (Figure 4A). After 20 min of adhesion, the extent of particle surface colonization, as reported by the mean number of bound bacteria per particle *n*_F_, was more than a hundred times higher than that of the curli-deficient mutant strain (Figure S4 and Figure 4A and 4B). In contrast, strain MG1655*gfp*Δ*csgA* exhibited single-phase kinetics characterized by a steady state corresponding to a low level of binding reached within the first seconds after particle contact (Figure 4B).

To quantitatively analyze these cell surface adhesion profiles, we considered the first adhesion phase ranging from 10 s to 10 min after particle/bacteria initial contact. Dynamics of early surface binding, *n*_F_(*t*) was adjusted to the first-order–like kinetics equation *n*_F_(*t*) *= N*_max_(1 – *exp*(*-k*_a_ *t*)) for both types of bacterial cells, with *N*_max_ the maximum mean colonization level displayed at plateau and *k*_a_ the apparent time constant of the adhesive process. The colonization plateau was obtained in both cases in the presence of a large fraction of free-floating cells.

For MG1655*gfp*Δ*csgA,* the steady state was characterized by an *N*_max_ value equal to 0.25 ± 0.3 bacteria per particle (bact part^−1^), which corresponded to the attachment of one cell for every four particles. This very restricted binding occurred rapidly after cell–particle contact, with an initial colonization rate reported by *N*_max_ *k*_a_ equal to 1.3 bact part^−1^ min^−1^. The rapid establishing of this low-level steady state (*t*_1/2_ = 0.13 min) indicated that cell and particle surfaces were essentially repulsive to each other under the conditions of the experiment, contrary to what could have been expected from opposite charge (negative cells and positive particles) surfaces. This point is further investigated below.

The curli producer strain MG1655*gfpompR234* displayed, in phase I, a ten times higher colonization level than the curli-defective strain (*N*_max_ = 3.2 ± 0.5 bact part^−1^) and exhibited a longer characteristic time (*t*_1/2_= 2.8 min and an initial binding rate of 0.8 bact part^−1^ min^−1^) (Figure 4A and 4B). This indicated that, in the presence of curli, cell and particle surfaces did not experience the repulsive potential observed between curli-defective cells and particle surfaces. Nevertheless, in this phase I, MG1655*gfpompR234* cells exhibited a colonization plateau in between three and four attached cells per particle, which represented a rather sparse surface occupation. This could be due to particle surface heterogeneity—of molecular nature or concentration—resulting in a low density of adhesive sites for bacteria in this first adhesive phase. Fresh particles added at the plateau to the cell–particle suspension displayed the same colonization kinetics as those initially present at *t* = 0 (Figure 4C), demonstrating that remaining floating bacteria still had the capacity to adhere to freshly added particles. On the other hand, no additional colonization occurred (stable *n*_F_) when additional bacteria were added at the colonization plateau (unpublished data).

These results suggest establishment, on the particle surface, of a repulsive potential that limits MG1655*gfp*Δ*csgA* adhesion but is partially overcome by the presence of curli adhesion to the surface of MG1655*gfpompR234.*

### Contact with the Cell Suspension Induces Rapid Surface Conversion That Modifies Initial Surface Properties and Colonization Rates

To investigate the possible origin of the repulsive potential, we monitored particle surface charge during colonization using the cationic dye PI labeling procedure (see above). As shown above (Figure S1), aminated particles suspended in PBS or M63B1 fresh medium did not adsorb the dye. Their FL3 intensity remained at the level of the background, corresponding to particles in the absence of dye. In contrast, when PI was added to the analysis test tubes taken from the incubation sample in the presence of exponentially grown bacteria, we measured a rapid increase in the PI labeling signal both with curli producer and nonproducer bacteria (Figure 4D). This indicated that the particle surface—initially positively charged—had turned into a negative surface potential, which enabled PI adsorption. This PI labeling affected both cell-free and colonized particles in the suspension (Figure S5A), suggesting that soluble species present in the bacterial supernatant were responsible for this effect. Consistently, aminated particles resuspended in filter-sterilized culture supernatants were labeled with PI, confirming production by bacterial cultures of anionic molecules. Furthermore, labeling intensity increased when supernatant stemmed from a longer overnight culture (unpublished data). No significant bacterial cell death was observed within the culture before or after incubation, as indicated by the absence of noticeable number of red-labeled bacteria on fluorescence microscopy images.

Indeed, in case of cell death, the dye contained in our samples would cross cell membrane and accumulate inside the cell through DNA association. Thus, only a small, naturally occurring bacterial lysis, which releases bacterial products in the medium, is expected to take place in our experiments, suggesting that this phenomenon could contribute only marginally to the surface properties changes observed. When COO^–^ particles were tested, we observed that PI labeling, initially high with such anionic surface functionalization, slightly decreased, suggesting that these surfaces were also modified by cell supernatant, which finally set the exposed surfaces to similar potentials (Figure S5B). Zeta potential measurements were performed on particles first incubated in bacterial culture media coming from cultures of the F plasmid-bearing strain and then suspended at 10% v/v in water for measurements. Value shifted for cationic particles from +36 mV in unspent medium (+45 in pure water) to −32 mV after 15 min incubation in 12-h culture spent medium. Anionic particles displayed slightly reduced negative charge, shifting from −65mV in unspent medium to −42 mV after medium conditioning, completely corroborating the behavior reported by dyes labeling. Very similar results were obtained with supernatant from MG1655 strain (unpublished data). This surface charge conversion to a negative potential was thus likely to explain the repulsive potential observed against bacteria.

To further investigate this, MG1655*gfpompR234* or MG1655*gfp*Δ*csgA* bacteria were placed in contact with both positively (NH_3_^+^) and negatively (COO^–^) charged particles, simultaneously and in the same sample. PI labeling enabled us to differentiate both types of particles within the sample (Figure S5C). For both strains, COO^–^ particle colonization was only slightly reduced compared to colonization on NH_3_^+^ particles, as if cells sensed very similar surfaces (unpublished data).

These results indicate that negative surface charge conversion induced by bacterial supernatants determines the surface potential of the interaction with cells independently of the initial surface state. In the absence of curli, this negative and thus repulsive potential dominates the interaction. Expression of curli at the cell surface obviously enabled overcoming this repulsion either by superimposing a much stronger binding force or by allowing the interaction to occur at a longer surface separation distance where the electrostatic potential had dropped. Indeed, in this high ionic strength medium, the Debye length is short and the electrostatic interactions quickly vanish with distance.

### A Second Colonization Phase Observed with Curli-Expressing Bacteria Was Correlated with an Increase in Free-Floating and Surface-Associated Bacterial Aggregates

**Figure 5:**
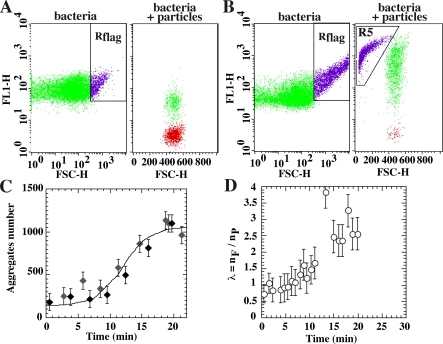
Self-Association of Free-Floating Cells and Cooperative Effects in Curli-Producing Bacteria Bacteria (left) and colloid (right) dot plots of MG1655*gfpompR234* brought into contact with NH_3_^+^ particles before (A) and after (B) second colonization phase onset. (C) Number of events comprised in the R_flag_ gate in the presence (black diamond) or absence (gray diamond) of particles. Cells were taken from a culture (DO = 0.5) and resuspended by pipetting in an adequate volume of buffer at time *t* = 0. (D) Time dependence of cooperative index, λ, during MG1655*gfpompR234* colonization.

After the initial binding phase, the MG1655*gfpompR234* colonization kinetics profile systematically displayed a sudden increase about 10–12 min after cell–particle contact (Figure 4A and 4E). Concomitantly, we observed a drastic increase in the population contained in the gate characteristic of bacterial aggregates (R_flag_), along with the appearance in the colloid plot of a new population collected in gate R5 (bacterial aggregates fluorescence (channel 1) region in particles records), corresponding to larger size particle-free bacterial aggregates (Figure 5A–5C). The kinetics curve showed that MG1655*gfpompR234—*but not MG1655*gfp*Δ*csgA—*aggregation started after a 10-min lag, suggesting that aggregation could result from a biological shift in curli production. Indeed, when cells were incubated 15 min in the presence of 100 μg/ml of the translation inhibitor chloramphenicol before adhesion test, the second adhesive phase was abolished as well as the drastic cell-cell aggregation increase (unpublished data). During this second phase, we also observed a rapid exponential increase in the cooperative index (Figure 5D), which indicated that already occupied particles were preferentially but not exclusively colonized. In addition, a synchronized increase in PI labeling of colonized particles (Figure 4D), but no change in cell-free particle labeling (unpublished data), showed that this second phase colonization induced an increase in the negative potential of the colonized surfaces.

Cell-cell aggregation also occurred when the experiment was conducted in the absence of particles, therefore ruling out the hypothesis of a surface-induced process (Figure 5C).

### Kinetics of Particle Colonization Is Dependent on the Nature of Adhesion Factors Expressed by Bacteria

**Figure 6:**
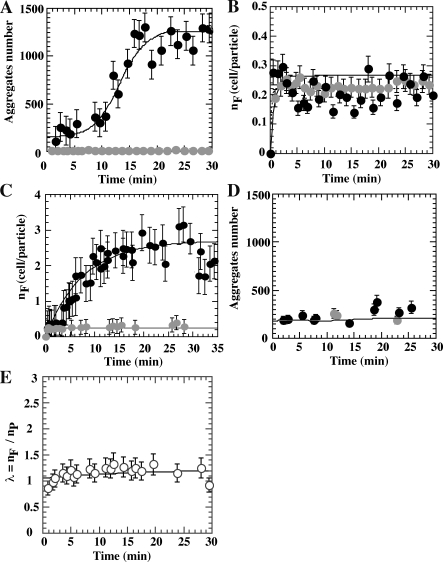
Aggregation and Surface Colonization Kinetics of Cells Expressing Different Cell Surface Adhesins Ag43 (A and B); F-pili (C and D) were examined under the same conditions as in Figure 4. (black circles) corresponds to cells producing indicated surface adhesin and (gray circles) to cells not producing surface adhesin. (E) Cooperative index in time for F pili–expressing cell colonization of NH_3_^+^ particles.

To evaluate the specificity of our observations with curli-expressing bacteria, we tested the adhesive properties of E. coli strains expressing other well-characterized adhesion factors. We first analyzed the adhesion profile of an E. coli strain that constitutively produces the autotransported adhesin Ag43, a short, 10-nm, surface-exposed adhesin known to promote cell-cell interactions and biofilm formation [12–14]. Ag43-mediated cell-to-cell association was confirmed by the presence of a significant number of events in aggregate-characteristic gates (Figure 6A). However, interestingly, the adhesion profile of Ag43^+^ cells (MG1655*gfp*PcL*flu*) was very close to that of the Ag43-depleted strain (MG1655*gfp*Δ*flu*) (Figure 6B)*.* This shows that, by contrast with curli expression, the strong cell-to-cell aggregation induced upon Ag43 expression did not correlate with surface attachment. This is probably due to lack of efficient initial bacterial adhesion enabling further anchoring of bacterial aggregates.

Next we examined the adhesive behavior of an E. coli strain expressing the F-conjugative pilus, which promotes both initial adhesion and biofilm maturation [15]. We compared colonization of the F-free strain (MG1655*gfp*) with an F-carrying strain (MG1655*gfp*F). We observed that F expression supported a significant one-phase surface attachment the kinetics of which could be approximated to a first-order-like process (Figure 6C). The plateau was obtained at *N*_max_=2.7 bact part^−1^ with a time constant *k*_a_ = 0.16 min^−1^, indicating an initial adhesion rate of *N*_max_ *k*_a_ = 0.45 bact min^−1^part^−1^, close to that of curli-expressing strain MG1655*gfpompR234* in phase I (see Figure 4C). Meanwhile, the F pilus-expressing cells did not exhibit significant self-association properties under these conditions (Figure 6D), indicating that this appendage did not support strong cell-to-cell interactions as curli or Ag43 did. Moreover, no second adhesive phase was observed and the cooperative index of F-carrying strains displayed no significant deviation from the unit (Figure 6E), which is consistent with the idea of a second adhesive phase correlated with both initial phase one association and strong cell-to-cell association.

Taken together, these results show that the kinetics of particle colonization are dependent on the nature of the expressed adhesion factor.

## Discussion

While bacterial adhesion genetics is being actively explored and produces increasing information on molecular factors that promote biofilm, the precise mechanism by which bacteria adhere to inert surfaces is not well understood [16,17].

We analyzed initial steps in bacterial attachment to abiotic surfaces by developing a new methodological approach that combined the use of a colloidal micrometric bead suspension as adhesion substrate and FCM. This strategy enabled the design of a real-time, multiparametric analysis to simultaneously monitor surface attachment, free-floating bacteria, and the surface physico-chemical state in the same sample, with the precision of a single cell and a time resolution of a few tens of seconds, without any separation step. Previous original attempts to assess initial binding kinetics used quartz microbalance equipment [18]. Although quartz resonance microbalance provides online monitoring of the physical state of the adsorbed cell layer reported by frequency shifts, it cannot be directly related to the numbers of attached cells. In contrast, FCM using colloids as adhesion substrates enabled us to directly monitor adhesion kinetics, providing initial binding rates that are unequivocal parameters of cell-surface association. These parameters are unaffected by bacterial features that are not part of initial adhesion, such as the division rate or resistance to shear or gene-dependent transition leading to biofilm. Moreover, the multi-parametric nature of FCM offered the opportunity of correlating initial adhesion with surface property changes and free-floating cell aggregation shifts, two phenomena involved in surface colonization. FCM kinetics are obtained on large cell populations with high statistical weight even for small subsets (>1%). However, unlike microscope imaging, this technique does not allow monitoring the fate of a single bacterial cell over time [19,20].

We showed that expression of several well-defined adhesion factors induced significant differences in early adhesion profiles recorded after cell surface exposure. We first demonstrated the direct implication of curli and F-conjugative pilus in cell surface attachment within the first minutes of contact. In contrast, the strain constitutively expressing short surface adhesin Ag43 displayed no significant rapid initial surface binding. Yet this appendage was implicated in abiotic surface colonization in various models [9,21]. This suggests that the Ag43 adhesin does not contribute to early steps in interactions between bacteria and the surface; rather, it may participate in subsequent steps of biofilm maturation not investigated in our short–time scale approach.

Kinetic characteristics of this initial surface binding support the hypothesis of the formation of a finite number of links between cell and particle surfaces rather than a physicochemical surface interaction, which would saturate either at cell population exhaustion or at surface overcrowding. Binding could occur either from the formation of a molecular complex between bacterial appendages and binding sites exposed at the particle surface after adsorption of surface active molecules produced by the cell suspension, or from hydrophobic interactions enabling cell strong adhesion on sparse and limited zones distributed over the particle surface. On the other hand, we show here, for all strains studied irrespective of the nature of exposed surface appendages, that surfaces undergo significant charge changes immediately when placed in contact with bacterial suspensions. The surface conditioning of host or environmental origin has been frequently addressed [2,22,23]. However, bacteria themselves produce macromolecules with sufficient surface activity to play a role in bacterial adhesion. This surface conversion induced by anionic surface-active molecules contained in the bacterial suspension could be one of the main reason why no clear relationship between initial surface physicochemical properties and bacterial adhesion has been established up to now [24], and that it might also partially explain why the DLVO theory has been generally unsuccessful in describing bacterial colonization.

We have no information as yet on the nature of the anionic biomacromolecule involved in the observed fast surface conversion, although bacterial surface polysaccharides such as released capsule fragment or LPS might be good anionic macromolecular candidates [25,26]. This surface conditioning accounts for the repulsive potential observed between MG1655*gfp*Δ*csgA* and the particle surface. This phenomenon is of particular interest in elucidating bacterial adhesion mechanisms, since it might constitute a mechanism by which nature selects the adhesive organisms, by first setting a repulsive surface potential.

In addition to initial surface contact, curli-expressing bacteria exhibited a second kinetic phase that correlated with the onset of significant bacterial self-aggregation. This second colonization phase could be due to the accumulation of curli subunits at the surface of the bacteria and developed cooperatively. Suppression of this second phase by chloramphenicol treatment known to stop translation suggests that de novo synthesis of curli during the adhesion process is responsible for curli accumulation (unpublished data). Consistently, when curli genes were placed under the control of an anhydrotetracyclin inducible promoter, ptetO [27], both aggregation and adhesion increased with increasing concentration of the inducer (unpublished data). Interestingly, although the strain constitutively expressing adhesin Ag43 displayed a strong aggregation phenotype, as expected from previous reports, it did not display secondary surface colonization [13,27,28]. Consistently, no second colonization phase was observed with E. coli expressing the F-pilus, which exhibited no rapid free-floating self-association in our experimental conditions using dispersed exponentially grown bacteria. Altogether, this demonstrates that early surface-bound cells could serve as anchors initiating cell associations, in good agreement with the role played by curli in biofilm maturation [10,29,30] and suggests that extensive cell-cell aggregation can amplify surface colonization, provided sufficient initial surface binding has occurred.

Many reviews on bacterial adhesion have contributed to spreading the notion of a general two-step mechanism comprising primary reversible adhesion, in which most bacteria leave the surface on-and-off to join the planktonic phase, followed by secondary irreversible attachment [2,20,22,31]. This so-called reversible-to-irreversible transition actually describes differential resistance to shear due to contact maturation in an out-of-equilibrium process. In experiments presented here, we monitored only irreversible adhesion in the thermodynamic sense; indeed no spontaneous binding shift upon dilution of suspension was observed. We show here that these irreversible interactions could take place during the first seconds of the contact independently of a prior reversible step. It is very likely that in our experiments, reversible events such as those described by Agladze et al. are instantaneously discarded by exposure to hydrodynamic flow (stirring and FCM shear flow) [32].

In conclusion, we have introduced a new short–time resolution tool for quantitative and statistical analysis of cell-surface adhesion. It enables determination of initial cell-surface binding kinetics and analysis of initial adhesive behaviors conferred by different bacterial cell surface structures. Beyond initial adhesion, we show that cell–cell aggregation properties held by several surface appendages amplify surface colonization once initial adhesion is established. This suggests a biofilm development scenario in which various adhesion factors contribute to different but complementary tasks to colonize the abiotic surface. During this process, micrometric structures such as curli and F-pilus support the formation of initial contact with the surface, the properties of which are strongly influenced by surface-active biomacromolecules. Further elucidation of molecular interactions behind initial steps in bacterial adhesion might help to elaborate original approaches to limiting biofilm development.

Finally, we believe that quantitative and kinetic dissection of early adhesion events could also represent a powerful way to investigate other aspects of biofilm development, including evaluation of potential antiadhesive compounds, gene expression upon surface contact or strain competition and cooperation during surface colonization.

## Materials and Methods

### Bacterial strains and growth conditions.

Constitutive curli producers (MG1655*gfpompR234*) were obtained by transducing, into *gfp*-tagged MG1655 (MG1655*gfp*), the *ompR234* mutation that specifies a gain of function allele of *ompR*, a gene encoding an activator of the curli operon [10]. MG1655*gfp*F carries a derivative of the F-conjugative plasmid. Non-curli producers (MG1655*gfp*Δ*csgA*), non-Ag43 producers (MG1655*gfp*Δ*flu*), as well as constitutive Ag43 (MG1655*gfp*PcL*flu*) producers were constructed by a three-step PCR procedure as described in [33,34]. The latter strains were constructed by introducing, in front of the *flu* gene, the *km*PcL cassette, which enables constitutive expression of chromosomal target genes [27]. Primers used to perform, verify, and sequence the different constructions are listed in Table S1. All strains were grown in lysogeny broth (LB) medium or, for adhesion experiments, in defined M63B1 medium with 0.4% glucose (M63B1Glu) at 30 °C for curli experiments or 37 °C for other experiments. Antibiotics were added when required: kanamycin (Km, 50 μg/ml), chloramphenicol (Cm, 25 μg/ml), ampicillin (Amp, 100 μg/ml), spectinomycin (Spec, 50 μg/ml), and tetracycline (Tet, 7.5 μg/ml).

### Colloidal surfaces.

10-μm-diameter polystyrene latex particles functionalized with carboxyl groups (COO^–^) were purchased from Polysciences. Particles were used either with their initial carboxyl functionalization after extensive washing and re-suspension in M63B1 minimal medium, or after surface treatment with polyethylenimine permethobromide a cationic, 6,300 molecular weight, branched polymer according to the principle initially introduced by Decher [35]. Briefly, particles were washed in pure water by filtration over 0.60 μm of diameter filter, adjusted to a concentration close to 10^6^/ml and gently stirred for 5 min in the presence of 3 × 10^−3^ M polyethylenimine (monomer concentration), which corresponds to an excess of positive charges compared to the negative charges of the colloidal particles. The permethylated amine polymer adsorbed rapidly onto the negatively charged particle, producing a net charge inversion and providing a cationic amino-functionalization (NH_3_^+^ particles). Particles were then washed four times before being concentrated five times in the experiment buffer, usually PBS or M63B1. The quality and stability of the deposit were checked by measuring the zeta potential in water using a Malvern zetasizer nano ZS Malverninstrument: −55 mV and +45 mV for COO^–^ and NH_3_^+^ particles, respectively. The deposit remained stable for several weeks, therefore confirming the robust adsorption already mentioned by other authors [36]. The colloid particles used in this study did not display any bactericidal activity against E. coli cells.

### FCM.

FCM analyses of bacteria and colloid suspensions were performed using a Becton-Dickinson flow cytometer (Facscalibur). GFP and green dye PYR emissions were recorded in fluorescence channel FL1 (band pass centered on 530 nm). The PI signal was collected in channel FL3 (>650 nm). Two different acquisitions were performed on each sample to collect either the bacterial signal or the colloid signal (lower FSC and SSC (side scattering) amplifications were used for colloids, but the same fluorescence gain settings were used for both objects). At least 1,500 events were recorded in colloid acquisition and 15,000 in that of bacteria. Data were analyzed using CellQuest (BDIS) and FlowJo (Tree Star) multivariate analysis software.

### Adhesion assay.

Cells and particles were brought into contact in a round-bottom tube. An adequate volume of exponentially growing bacterial suspension—DO_590_ = 0.5–0.6 in M63B1Glu—was usually injected at time *t* = 0 into a particle suspension adjusted to the appropriate concentration in M63B1 and stirred at room temperature on a soft vortex (1,000 rpm min^−1^). Total volume of this incubator was usually equal to 500 μl. Aliquots of 5–20 μl were taken at given incubation times for immediate analysis in FCM in 300 μl PBS supplemented with 5 picomoles PI or microscope imaging.

## Supporting Information

Figure S1 Surface Charge Detection by FCM FCM dot plots of PYR- (2nd column) and PI- (3rd column) labeled cationic (1st row) and anionic (2nd row) particles. Five picomoles of the appropriate dye were introduced in each particle sample.
(491 KB PDF)

Figure S2 GFP^+^ and GFP^–^ E. coli Mixture in FCM Analysis Fluorescence dot plots of GFP^+^ and GFP^−^ cell mixtures 50/50, 10/90, and 1/99 from left to right. Aliquots of exponentially growing cultures were diluted in PBS to obtain a 5 × 10^6^ cells/ml suspension. Numbers of cells in each population very closely matched proportions expected from the mixtures.
(451 KB PDF)

Figure S3 Fluorescence Signal Calibration FCM signal of particles alone (A) or cell-particle contact using 100-fold excess of particles over bacteria (B), ensuring that only rare but single cell adhesion events would statistically appear compared with the signal of free-floating bacteria, *fl*_i_, recorded in parallel on the same sample (C). Mean fluorescence (FL1) intensity of single cell–carrying particles, calculated on ten independent recordings (72 single cell–carrying particles detected), was found equal to 42 ± 5, whereas free-floating bacteria displayed a mean fluorescence of 41 ± 3, calculated for 98% of the population around the distribution mode (FL1). An example of data is shown here. Gate R2 corresponds to bare particles, whereas gate R3 corresponds to colonized particles.
(462 KB PDF)

Figure S4 MG1655*gfpompR234* and MG1655*gfp*Δ*csgA* Surface Colonization FCM dot plots and kinetic curves of MG1655*gfpompR234* (A) and MG1655*gfp*Δ*csgA* (B). Particles and cells were brought into contact at time *t* = 0. Plots show net segregation between cell-bearing particles and free particles. Cell to particle ratio was around 200 and particle concentration equal to 6 × 10^6^/ml.
(500 KB PDF)

Figure S5 Surface Charge Inversion Effects (A) FL1 and FL3 dot plots showing red labeling of both colonized and cell-free particles before (2 left panels) and from 1 min after (3 right panels) cell–particle contact for MG1655*gfpompR234* and NH_3_^+^ particles. We introduced 5 picomoles of PI in all analysis samples and collected mean particle fluorescence in channel 3 (FL3) corresponding to PI emission (>670 nm).
(B) Kinetics of surface conversion for both cationic (█) and anionic (•) particles.
In panel (C), parallel colonization of anionic and cationic particles within the same sample tube is shown. From left to right: scattering, FL3 and FL1 signals of particle mixture brought into contact with MG1655*gfpompR234* cells for 5 min. Gate R7 (particles high fluorescence intensity (channel 3) region) comprises COO^–^ particles with their high PI labeling; R8 (particles low fluorescence intensity (channel 3) region) includes NH_3_^+^ particles.
(640 KB PDF)

Table S1 Primers Used in This Study (38 KB DOC)

## Abbreviations

FCM: flow cytometry
FL1: mean fluorescence intensity in channel 1 (BP530/15nm)
FL3: mean fluorescence intensity in channel 3 (LP650nm)
*fl_i_*: bacterial cell unitary fluorescence in FL1
FSC: forward scattering
GFP: green fluorescent protein
PBS: phosphate-buffered saline
PI: propidium iodide
PYR: pyranine
R1: free particles scattering region
R2: free particles fluorescence (channel 1) region
R3: bacteria-associated particles fluorescence (channel 1) region
R_b_: bacteria scattering region
R_flag_: bacterial aggregates fluorescence (channel 1) region in bacteria records
R_flb_: bacteria region
SSC-H: side scattering height
