# Supplementary material for: A Short–Time Scale Colloidal System Reveals Early Bacterial Adhesion Dynamics
Source: PLoS Biol. 2008 Jul 8;6(7):e167. doi: 10.1371/journal.pbio.0060167 (PMC2443189; doi:10.1371/journal.pbio.0060167)
Supplement: Supplementary file 1 [file pbio.0060167.sg001.pdf]

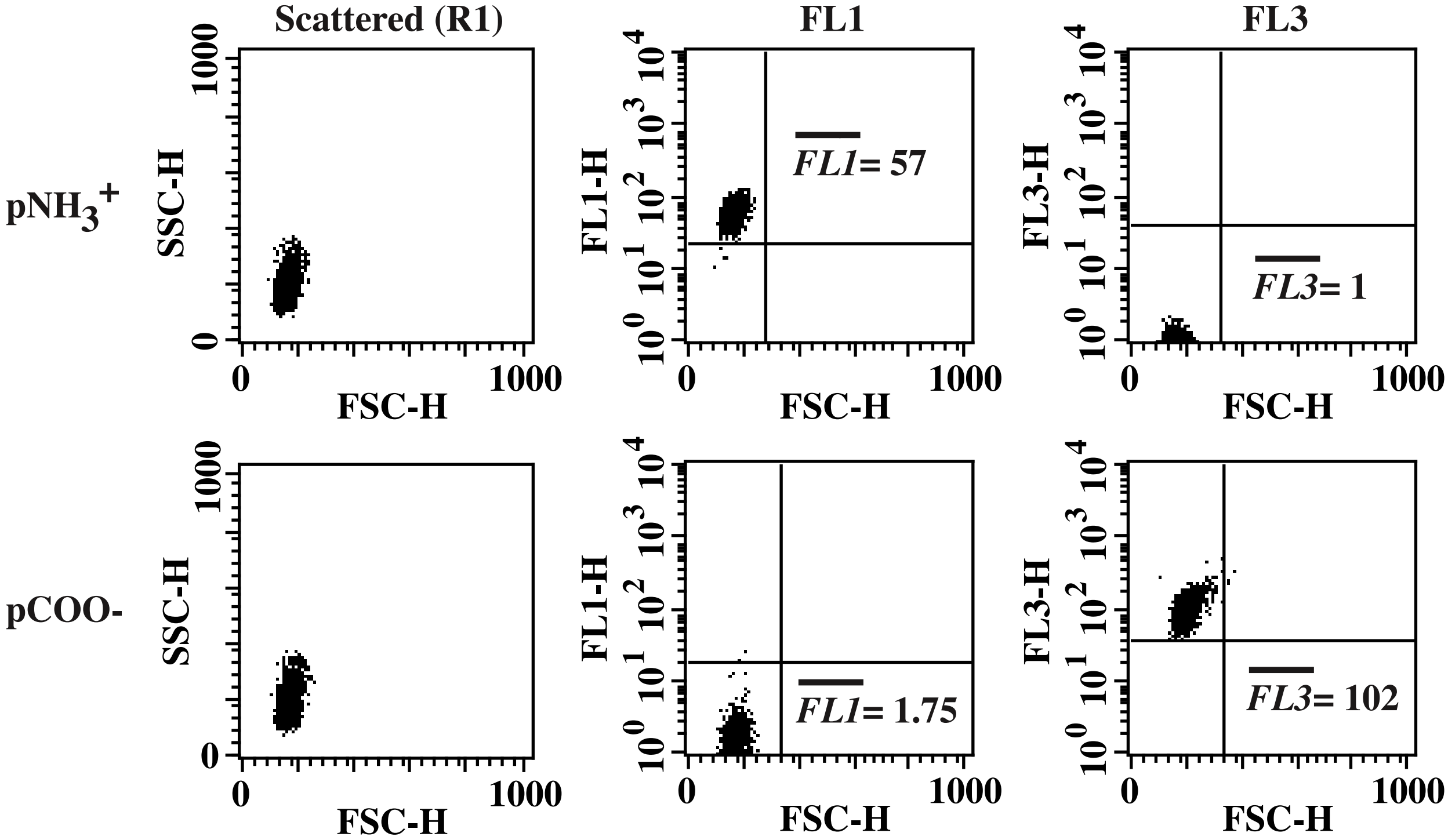
Scattered (R1)
FL1
FL3
pNH3+
pCOO-
SSC-H
FSC-H
FL1-H
FL3-H
0
1000
FL1= 57
FL3= 1
FL1= 1.75
FL3= 102
