# Supplementary material for: A Short–Time Scale Colloidal System Reveals Early Bacterial Adhesion Dynamics
Source: PLoS Biol. 2008 Jul 8;6(7):e167. doi: 10.1371/journal.pbio.0060167 (PMC2443189; doi:10.1371/journal.pbio.0060167)
Supplement: Supplementary file 2 [file pbio.0060167.sg002.pdf]

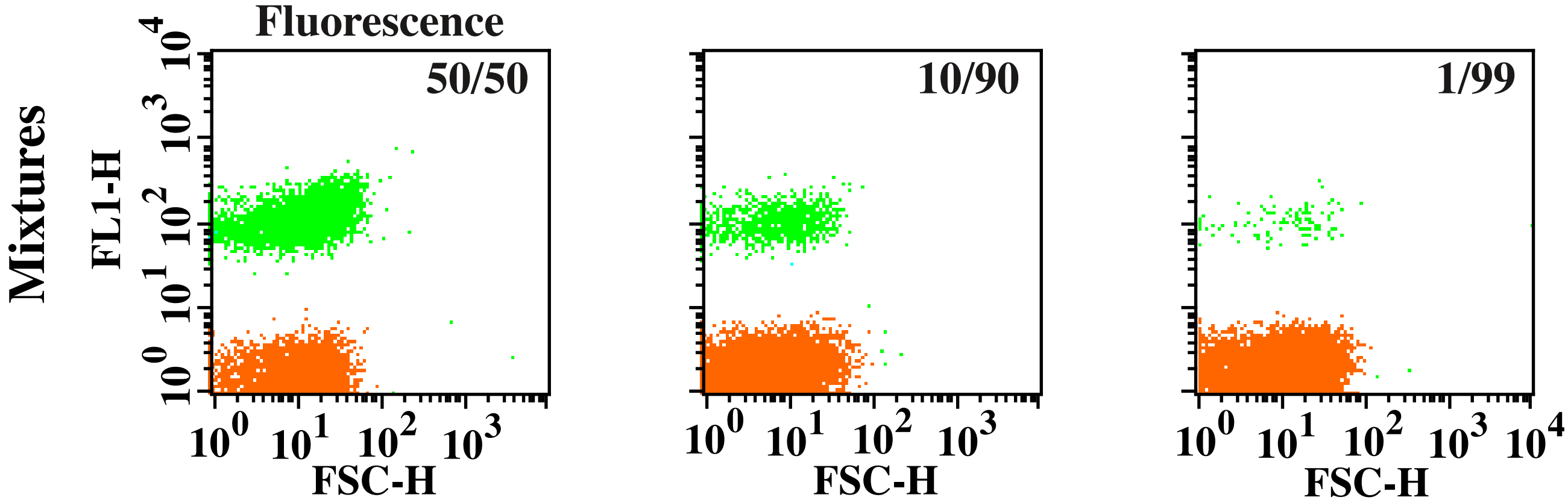
Fluorescence
Mixtures
50/50
10/90
1/99
FL1-H
FSC-H
FSC-H
FSC-H
