# Supplementary material for: A Short–Time Scale Colloidal System Reveals Early Bacterial Adhesion Dynamics
Source: PLoS Biol. 2008 Jul 8;6(7):e167. doi: 10.1371/journal.pbio.0060167 (PMC2443189; doi:10.1371/journal.pbio.0060167)
Supplement: Supplementary file 3 [file pbio.0060167.sg003.pdf]

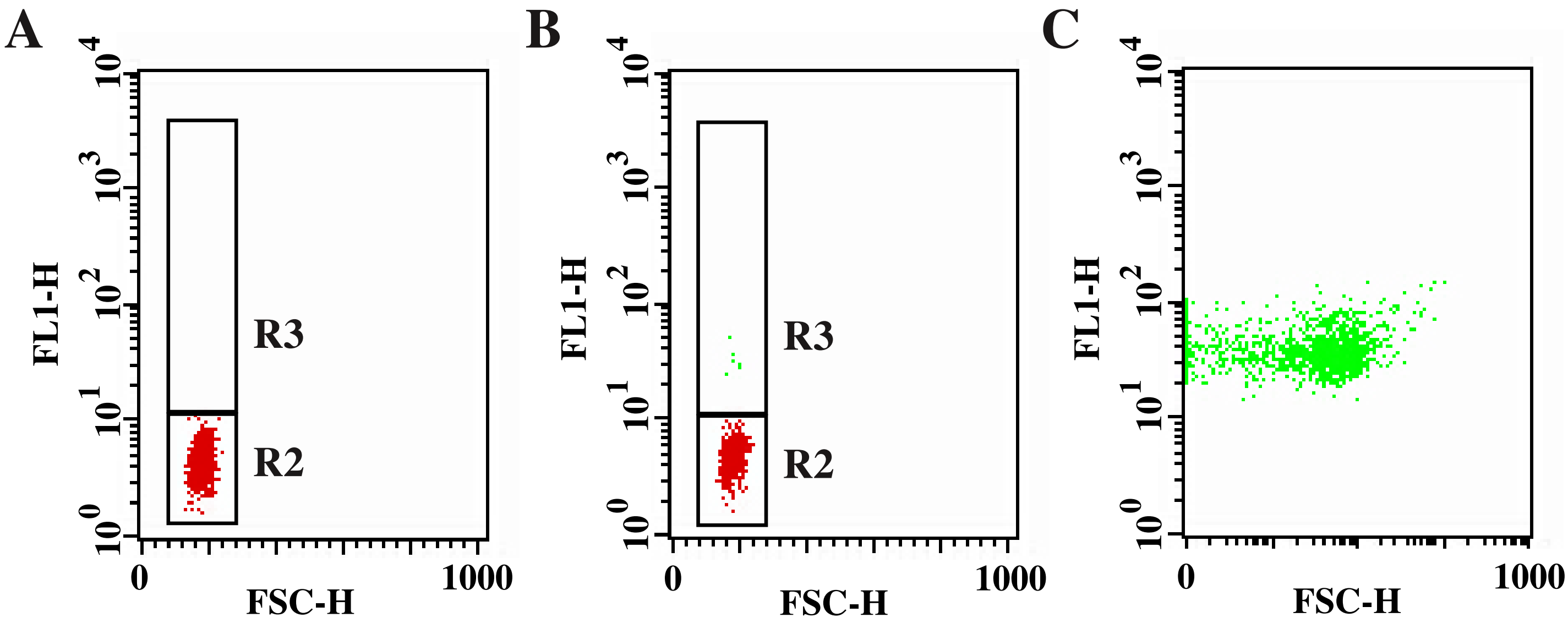
A
B
C
FL1-H
FSC-H
R3
R2
10^0
10^1
10^2
10^3
10^4
0
1000
