# Supplementary material for: A Short–Time Scale Colloidal System Reveals Early Bacterial Adhesion Dynamics
Source: PLoS Biol. 2008 Jul 8;6(7):e167. doi: 10.1371/journal.pbio.0060167 (PMC2443189; doi:10.1371/journal.pbio.0060167)
Supplement: Supplementary file 4 [file pbio.0060167.sg004.pdf]

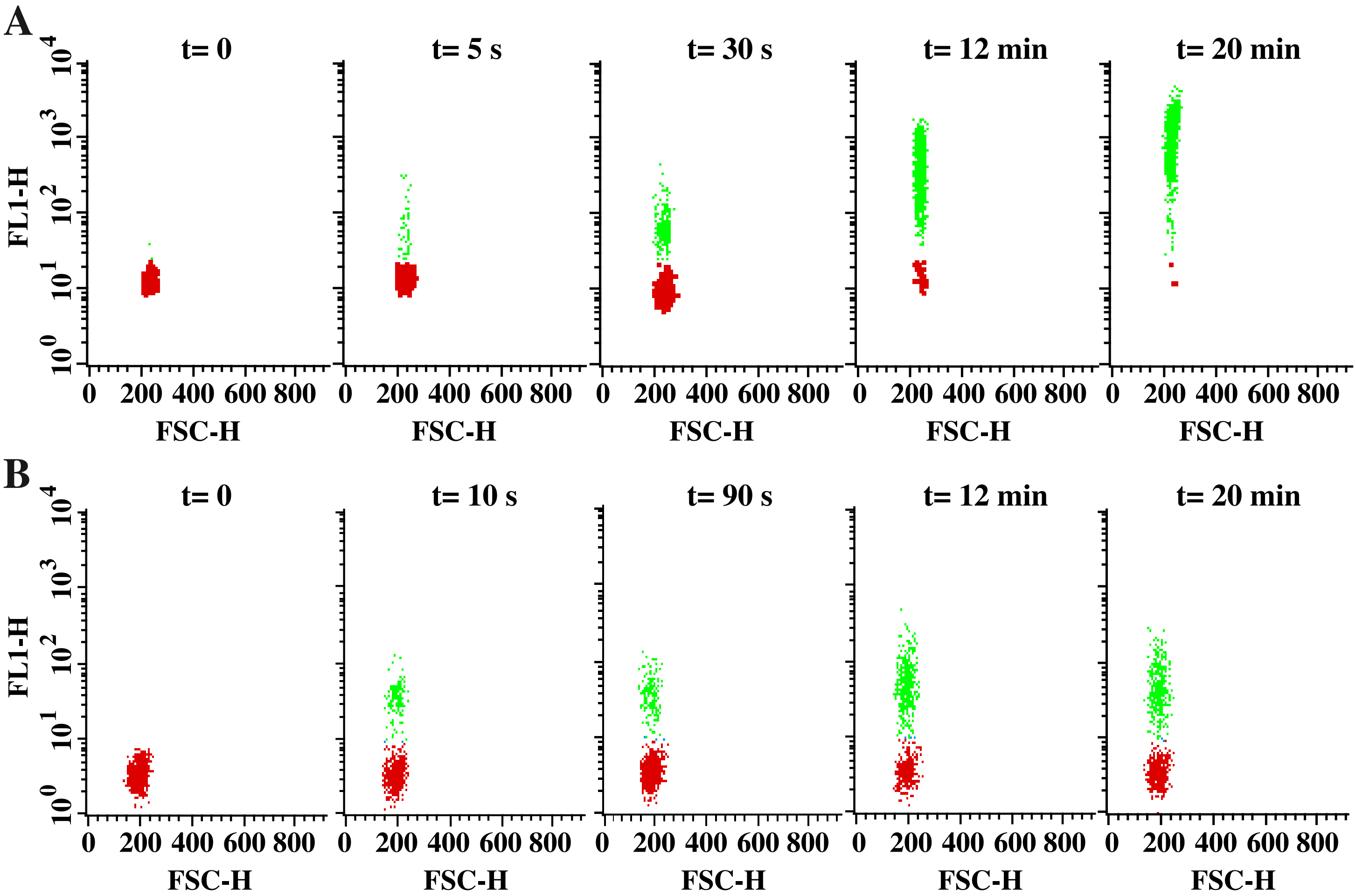

A
t= 0
t= 5 s
t= 30 s
t= 12 min
t= 20 min
FL1-H
FSC-H
B
t= 0
t= 10 s
t= 90 s
t= 12 min
t= 20 min
FL1-H
FSC-H
