# Supplementary material for: A Short–Time Scale Colloidal System Reveals Early Bacterial Adhesion Dynamics
Source: PLoS Biol. 2008 Jul 8;6(7):e167. doi: 10.1371/journal.pbio.0060167 (PMC2443189; doi:10.1371/journal.pbio.0060167)
Supplement: Supplementary file 5 [file pbio.0060167.sg005.pdf]

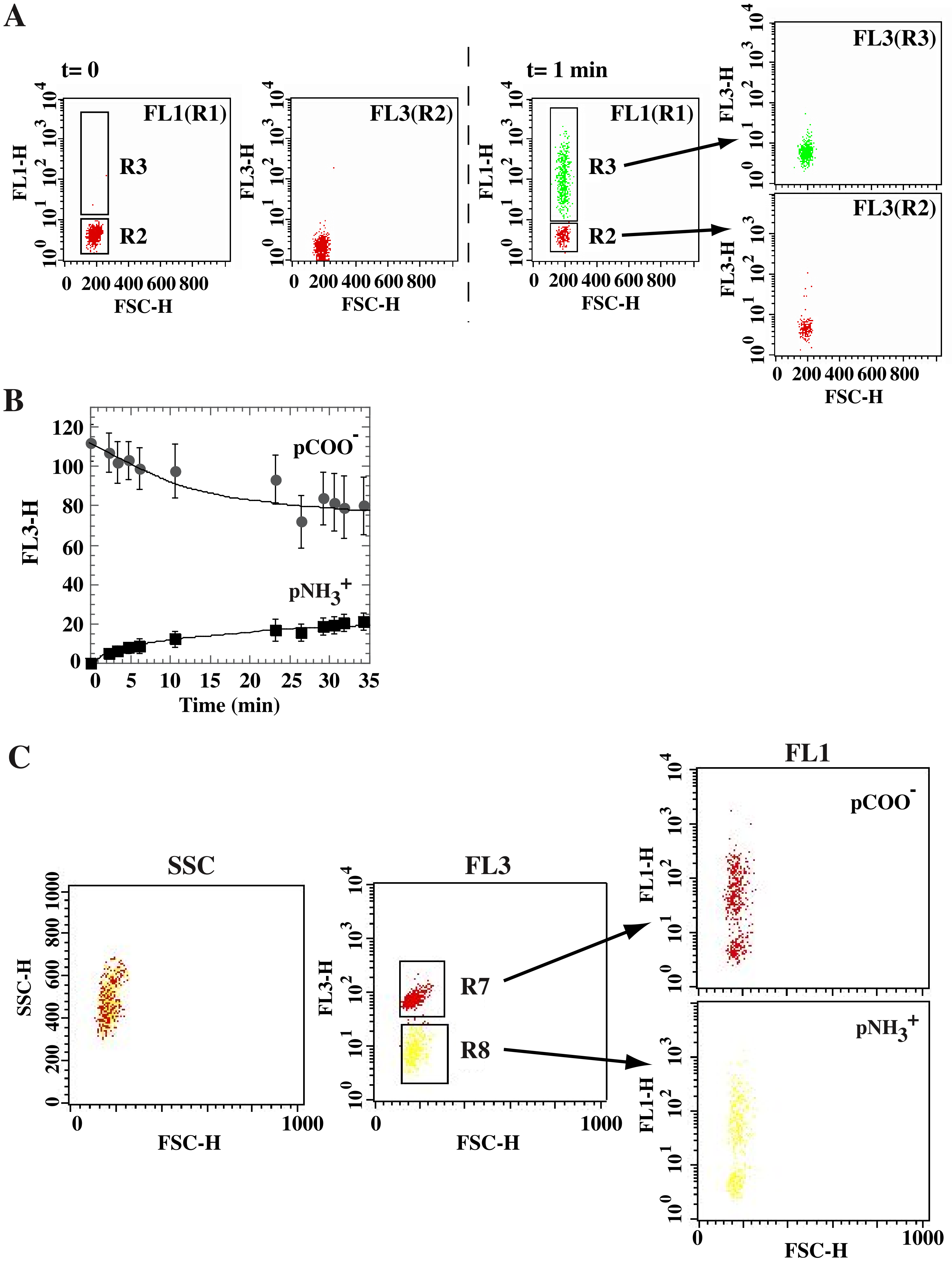
A
t= 0
FL1(R1)
R3
R2
FL1-H
FSC-H
FL3(R2)
FL3-H
t= 1 min
FL3(R3)
0 200 400 600 800
B
pCOO-
pNH3+
FL3-H
Time (min)
120
100
80
60
40
20
0
5
10
15
20
25
30
35
C
SSC
SSC-H
FL3
R7
R8
FL1
pCOO-
pNH3+
FL1-H
FSC-H
1000
