# Supplementary material for: A Short–Time Scale Colloidal System Reveals Early Bacterial Adhesion Dynamics
Source: PLoS Biol. 2008 Jul 8;6(7):e167. doi: 10.1371/journal.pbio.0060167 (PMC2443189; doi:10.1371/journal.pbio.0060167)
Supplement: Supplementary file 6 [file pbio.0060167.st001.doc]

**TABLE S1.** Primers used in this study.

| **Primer name** | **Sequence** |
| --- | --- |
| Primers used to generate the ∆*csgA*::*aadA7* (Spec^R^) mutant | |
| csgA.A1.500-5 | GATGTTAACAATACTGGGTG |
| csgA.B1.500-3 | gggttgtgttatccatactttc |
| csgA.B2.Aad7.Lnp-5 | CTGCTTGGTGCCAGCCAATGATAAtacatcatttgtattacag |
| csgA.A2.Aad7.L-3 | GTATTTATTCGGCGcaattgCATgtaaaacccccatcggattg |
| csgA.ext-5 | cgcagcgttaacaaaatacagg |
| csgA.ext-3 | caaactgccgagcataagag |
| Primers used to generate the ∆*flu*::*km* (Km^R^) mutant | |
| flu.A1.500-5 | CCCgAATTCTgCggTggACCggATATTTg |
| flu.B1.500-3 | atttccttgctgatatcttc |
| flu.B2.GB.L-5 | cttcacgaggcagacctcagcgccTAAcagaaccatcgcctctctgtg |
| flu.A2.GB.L-3 | gattttgagacacaacgtggctttCATcagcttttccttagattg |
| flu.ext-5 | atacgctggtcagtgcgctc |
| flu.ext-3 | atcagtgacggtgaaatatc |
| Primers used to generate the *km*PcL*fim* (Km^R^) strain | |
| fimA.insA1.500-5 | CCCgAATTCTgCggTggACCggATATTTg |
| fimA.insB1.500-3 | TGGAACGGAATGGTATTGGTTC |
| fimA.PcL.L-5 | CGGTGATAATGGTTGCATGTACTAagttttttgaaaggaaagcagcAT |
| fimA.PcL.L-3 | GTGAGAATTACTAACTTGAGCGAAcaaaatttaatattcgttcaacta |
| fimA.ins.ext-5 | cagacgaacacttgcggtggtc |
| fimA.ins.ext-3 | TTGAAGGTCGCATCCGCATTAG |
| Primers used to verify cassette insertions and to sequence constructions | |
| spec.verif-5 | CCGATACTCTGAAGCTATGGAACTC |
| spec.verif-3 | ACAAATGCACGGCCAGCAAC |
| kmGB-verif-5 | GTTTTCTCCTTCATTACAGAAACG |
| kmGB-verif-3 | ATATGGCTCATAACACCCCTTG |
| PcL-km-verif-5 | CAGAGCAGCCGATTGTCTGTTG |
| PcL-km-verif-3 | CTTCCTCGTGCTTTACGGTATCG |
